# Special Challenges of Sensitive Images: A HIMSS-SIIM Enterprise Imaging Community Whitepaper

**DOI:** 10.1007/s10278-024-00980-8

**Published:** 2024-02-13

**Authors:** Alexander J. Towbin, Delaney D. Ding, Moneif Eid, Heather Kimball, Julia Komissarchik, John Memarian, Seetharam C. Chadalavada

**Affiliations:** 1 Department of Radiology, Cincinnati Children’s Hospital, 3333 Burnet Ave, MLC 5031, Cincinnati, OH 45229 USA; 2 Department of Radiology, University of Cincinnati College of Medicine, Cincinnati, OH USA; 3 University of Florida College of Medicine, Gainesville, FL USA; 4 Ministry of Health, Riyadh, Saudi Arabia; 5 Stanford Health Care, Palo Alto, CA USA; 6 Glendor Inc, Draper, UT USA; 7 Imaging & Informatics, CitiusTech, Indianapolis, IN USA; 8 Department of Radiology, University of Cincinnati College of Medicine, Cincinnati, OH USA

**Keywords:** Enterprise imaging, Sensitive images, Patient safety, Medical photography, Medical imaging

## Abstract

Sensitive images represent a new challenge in enterprise imaging. These images, often containing nudity or gruesome content, have the potential to cause emotional harm to patients and people who view the images. Unfortunately, the interoperability standards used in imaging informatics have not yet addressed this issue. Because of this, the software solutions used in healthcare information technology are not able to offer patients and other viewers of image protections. In this Health Information Management Systems Society (HIMSS)/Society for Imaging Informatics in Medicine (SIIM) Enterprise Imaging Community Whitepaper, we define sensitive images, identify unique challenges related to their management, and provide recommendations for future solutions to protect our patients.

## Introduction

The widespread adoption of smart devices with high-quality digital cameras has made medical photography feasible at a large scale. These photographs, when indexed, managed, stored, distributed, and viewed across the enterprise, enhance the electronic health record [1]. The Health Information Management Systems Society (HIMSS)/Society for Imaging Informatics in Medicine (SIIM) Enterprise Imaging Community Enterprise Imaging Community has previously described some of the key aspects of visible light imaging and medical photography [2]. This prior work defined terms related to photodocumentation and provided a general overview of the key challenges facing clinical photography. In that white paper, sensitive images were described but not discussed in detail. Based on feedback received from patients, providers, and healthcare organizations, the HIMSS-SIIM Enterprise Imaging Community believes that the challenges of sensitive images require more attention. Thus, the purpose of this white paper is to define sensitive images, identify their unique challenges, and provide recommendations for future solutions. Throughout the whitepaper, short clinical vignettes will be provided to highlight specific issues.

## Defining Sensitive Images

Oxford Languages defines sensitive content as “anything that may cause offence to a reader or user, particularly in relation to religion, race, gender, politics, sexuality, disability, or vulgar language [3].” We believe that “may” is the key word in this definition as it implies that the reader is the one who decides whether the content is sensitive, not the author. Using the Oxford Languages definition as a guide, we define “sensitive images” as *any image obtained or used for the purpose of providing care with the potential to reveal personal information (beyond protected health information) that may cause the patient, patient proxy, or health care provider embarrassment, shock, grief, or emotional distress*. At the outset, we note that while healthcare providers may be used to seeing these images, they still may experience the same emotions felt by patients. Thus, they are included in the definition. There are several types of images that are generally considered to be sensitive.

### Nudity

#### Clinical Use Case #1

*A 53-year-old woman has newly diagnosed breast cancer. A breast surgeon takes photographs of the woman’s breasts before and after mastectomy and reconstructive breast surgery.*

#### Clinical Use Case #2

*A 27-year-old man with Poland syndrome is photographed during an ambulatory chest wall deformity clinic visit. The patient suffers from body dysmorphic disorder due to his chest wall deformity and does not want to see photographs of himself. The photographs are tagged as containing nude content based on institutional policy.*

In the initial visible light imaging whitepaper, the HIMSS-SIIM Enterprise Imaging Community stated that images of body parts covered by undergarments (such as in Clinical use case #1) are generally considered to be sensitive [2]. The prior whitepaper highlighted that this definition is based on cultural and societal norms and may differ by locale or between individuals with different sensibilities. For example, a photo of a bare-chested male is within societal norms in the USA. However, Clinical use case #2 highlights an example where a socially acceptable photograph may still be considered sensitive.

We note that radiologic images may be considered sensitive by this definition. We believe that this classification may be appropriate. Mammograms, radiographs of the pelvis, and CT examinations all provide images of sensitive body parts. In the case of a CT examination, 3-dimensional surface rendering reconstructions can be created from the source imaging data. Patients would undoubtedly consider these imaging studies to be similar to photographs of their nude body. Organizations will ultimately decide whether they should apply sensitive tags to radiologic imaging.

### Gruesome Images

#### Clinical Use Case #3

*A 25-year-old man suffered an open fracture of the femur in a car accident. Paramedics at the scene photographed the injury at the scene and sent it to the trauma hospital to prepare the team for the extent of the injury.*

Gruesome photographs have been defined as “images which are bloody, gory, or violent” [2]. This type of image may be difficult to identify, as its sensitive nature is dependent on the image’s context and the experience of the person viewing the image. Common types of gruesome images include trauma photos, surgical photos, gross pathology images, and wound photos.

### Forensic Images

#### Clinical Use Case #4

A 19-year-old woman was assaulted. Photographs of her face, chest, and abdomen were obtained, documenting her injuries.

Forensic images are used to document a potential crime. These images may or may not contain nudity. However, even when they do not contain nudity, their presence may cause severe emotional distress to the patient or other victims of a similar crime.

### Identifiable Images

#### Clinical Use Case #5

*A 12-year-old male with neurofibromatosis type 1 was seen by an ophthalmologist. Photographs of the iris were obtained to document Lisch nodules.*

Some organizations classify personally identifiable images as sensitive. Examples include photographs of the face, fingerprints, or retinas. By definition, these images constitute protected health information [4]. Organizations that include these images in their sensitive photo policy do so because they want to apply an extra level of security to the images. However, we do not believe that this type of image truly meets the definition of a sensitive photo. Instead, we believe that these images should be defined as private and protected under privacy regulations such as the Health Insurance Portability and Accountability Act of 1996 (HIPAA) in the USA or the General Data Protection Regulation (GDPR) in Europe.

## Tagging/Labeling Sensitive Images

### Clinical Use Case #6

*A 57-year-old man with a history of Crohn disease and perianal and genital fistulas presents to an otolaryngologist with a nasal mass. Photographs of the patient’s anus and external genitalia were obtained as part of his Crohn disease care. These are tagged as containing nudity. Other non-sensitive photographs obtained during colonoscopy and histopathology are included as part of this condition. New photographs of the nasal mass were obtained during the visit with the otolaryngologist. The nasal photographs are not considered to be sensitive. As the patient seeks a second opinion related to the nasopharyngeal carcinoma diagnosis, he does not want the sensitive photos related to his Crohn disease care shared with the new otolaryngologist. However, when the patient seeks a second opinion for care of his Crohn disease, he would like the new gastroenterologist to see these sensitive images.*

### Clinical Use Case #7

*A 62-year-old man with type II diabetes presents to the emergency room with Fournier gangrene. The Emergency Medicine physician obtained a photograph of the genital wound. The photographs are tagged as containing nudity and gruesome content.*

### Clinical Use Case #8

*A 32-year-old woman with cholecystitis undergoes cholecystectomy. Photographs of the abdominal incision are obtained during the healing process. The woman considers the images of her bare abdomen to be sensitive and asks that a nudity tag be applied to the photographs. Because she was still under the effects of anesthesia, her preference was not noted for the first photograph.*

### Clinical Use Case #9

*A 15-year-old girl is seen by a gynecologist for clitoromegaly and an orthopedic surgeon for scoliosis. The gynecologist obtained photos of the girl’s external genitalia to document her condition. The orthopedic surgeon obtained multiple scoliosis radiographs throughout the course of care. During the most recent visit, the orthopedic surgeon was reviewing the scoliosis radiographs with the girl and her mother. While opening studies, the surgeon accidentally opened the gynecologic images causing the patient emotional distress and the surgeon embarrassment. While the patient’s mother was initially involved in her child’s care, the patient has since expressed that she would prefer her mother not see her genitalia during clinic visits.*

While we have defined sensitive images, there are challenges with systematically applying a tag to identify an image as sensitive. This is predominantly a limitation of the Digital Imaging and Communication in Medicine (DICOM) standard. In its current form, the DICOM standard does not have a sensitive tag that can be applied at the study level. The only sensitive tag that exists is ParticipantObjectSensitivity [5]. This tag is set at the patient level and is used to protect celebrities or patients with specific conditions (such as human immunodeficiency virus infection). It does not provide the granularity needed to distinguish between different types of imaging studies (Clinical use case #6).

Integrating the Healthcare Enterprise (IHE) has created the Cross-Enterprise Document Sharing (XDS) profile. Within the profile, the Basic Patient Privacy Consent (BPPC) and Advanced Patient Privacy Consent (APPC) provide a framework to tag sensitive imaging studies [6, 7]. While BPPC and APPC provide some ability to identify imaging studies as sensitive, we do not believe that the profile is sufficient as permissions are defined by patient consent. Labels to identify the type of sensitive content (Clinical use cases #1–4) do not exist.

Ultimately, we believe that DICOM and IHE should create metadata fields that allow imaging studies to be tagged as containing nudity, gruesome content, and/or forensic content. We note that multiple labels may need to be applied to the same content (clinical use case #7). Additionally, other labels may be needed (Clinical use case #5) as the enterprise imaging continues to evolve.

If these fields were to exist, we believe that many labels could be applied in an automated manner. For example, the nudity tag could be applied automatically based on the body part imaged (e.g., images of the external genitalia, buttocks, or chest). Organizations could modify this list based on standard body part anatomy labels [8]. Because patients could select the anatomy from a body part map within the patient portal, the sensitive tag could be applied to images provided by patients in addition to those captured by healthcare practitioners. Other tags could be applied based on specialty, procedure description, or imaging location. Examples are included in Table 1.

**Table 1:** Examples where sensitive imaging tags could be applied in an automated manner

Sensitive tag	Tag triggered by	Example
**Nudity**	Body part	Photograph of the external genitalia
	Specialty	Photograph obtained by a gynecologist
	Procedure description	Colposcopy photograph
**Gruesome**	Specialty	Wound care photograph
	Procedure description	Gross pathology photograph
	Location	Operating room
	Application entity title or station name	Pathology grossing station camera
**Forensic**	Specialty	Coroner
	Procedure description	Autopsy
	Application entity title or station name	Coloposcopy camera

While most sensitive tags could be applied in an automated manner, it is important to note that healthcare providers should always have the added option to flag an imaging study as sensitive (Clinical use case #8). This manual option will allow providers to consider the sensitivities and wishes of their patient. Patients should also have this option for images uploaded through the patient portal.

Until changes are made to the DICOM standard and XDS profile, we suggest automatically prepending the procedure description with the word *sensitive* or a similar label. Some institutions may choose to provide additional clues to the nature of the content within the procedure description. Examples of this could include adding the letter N, G, or F after *sensitive* to reflect nudity, gruesome, or forensic content. As described above, this addition to the procedure description can be automated based on the triggers identified in Table 1.

We believe that there are several advantages to the automatic application of a sensitive label within the procedure description. First, the word *sensitive* can serve as a warning, allowing users to recognize the nature of the content before opening and viewing the imaging study (Clinical use case #9). Second, applying a label in this manner will allow organizations to understand how many visible light imaging studies contain sensitive content. For example, one organization identified 60% of their photographs as sensitive (unpublished data from Alexander Towbin, MD; data accessed May 2022). Third, using the procedure description to label sensitive images will enable later application of a sensitive content tag within a standard metadata field. When a standard-based approach is available, this already-archived content will be easily identified and the new tag applied. Finally, using the word *sensitive* within the procedure description will allow vendors to build some early rule-based protections for patients and other end users. For example, a vendor could create a pop-up alert warning the user that they are about to open sensitive content. The user would then have the choice of opening the study or moving on without opening it.

We recognize that there is a disadvantage to adding the word *sensitive* to the procedure description before a standard-based tag is applied. Namely, this label can be used to enable criminal behavior. Using this method of labeling studies, any user can query for, identify, view, and possibly download large volumes of sensitive content. We encourage vendors to create smart-auditing strategies to alert organizations to this behavior. Because this type of auditing does not yet exist, we also encourage organizations to develop and implement a routine sensitive images auditing strategy.

In addition to adding the word SENSITIVE to the procedure description, other stop-gap strategies can be applied at image capture to help protect patients. For example, an image capture application could add a black image with the words “SENSITIVE CONTENT” as the first image of any sensitive imaging study. This would prepare users for the content contained within the imaging study before it is viewed. Organizations should encourage their vendors to apply this type of creative solution until a standards-based approach can be adopted.

## Access and Permissions: Providers

### Clinical Use Case #10

*A 28-year-old woman is involved in a motor vehicle collision. Due to the accident, she has extensive facial fractures and lacerations. Photographs of her face are obtained in the trauma bay and are tagged as gruesome. The photographs appear as a relevant prior study to the radiologist interpreting the head CT. The radiologist would prefer a warning before these images are displayed. Later, after healing, the patient does not want to view these images of herself.*

A standard-based approach to identify and tag sensitive images is not enough. These standards must be used by vendors to create solutions, by organizations to set policies and apply permissions, and by individuals to define their preferences.

Ultimately, we believe that vendors must develop solutions that enable organizations to apply tiered access permissions within their applications. Access and potential restrictions may vary based on the role of the user and the type of sensitive content. This is described in more detail in the previous Visible Light Imaging Whitepaper [2]. Key concepts are highlighted below.

Within organizations, there may be multiple tiers of access permission for images containing nudity and forensic content. These could include:

1. Open access: the user can view the content without restriction.
2. Limited access: the user can view the images after entering their password or another type of access validation. This type of access provides a higher level of logging for audit purposes.
3. Restricted access: the user is unable to view the images.

In the ideal state, access rules could be applied based on descriptors such as provider specialty or provider relationship to the patient. For example, to address the scenario described in Clinical use case #9, the organization may determine that the gynecologist should have open access to view all sensitive gynecology images. In contrast, the orthopedic surgeon may have either limited access or restricted access to this same content. The organization may create an additional access rule providing all members of the patient’s care team with limited access to all content outside of their specialty and open access within their specialty. If this were to occur, the orthopedic surgeon providing care to the patient in Clinical use case #9 would be able to view the sensitive gynecology images after entering their password while their colleague (who is not a part of the patient’s care team) would not be able to view the images.

Different healthcare practitioners will have a different emotional reaction to seeing the same sensitive image. Thus, while access to images may be defined at the organization level, end users should have the ability to define their own preferences based on their own sensibilities. For example, while a user may have open access to view gruesome images, the user may define their preferences so that gruesome images are displayed using limited access (Clinical use case #10). We believe that this enhanced access preference will help to protect providers from opening a sensitive imaging study before they are prepared to view the content. If these user preferences exist, we would encourage vendors to allow flexibility in the creation of rules. For example, a user may wish to view surgical photos with open access but wound care photos with limited access.

## Access and Permissions: Patients

### Clinical Use Case #11

*A 72-year-old woman with multiple medical conditions requiring care provides her son proxy access to her medical record. Photographs were obtained before and after mastectomy and breast reconstruction.*

### Clinical Use Case #12

*A 3-year-old boy drowns in a neighborhood pool. An autopsy is performed during which photographs are obtained and tagged as gruesome. When the autopsy report is filed, the parents receive an alert from the patient portal stating that a new note is available. The parents click the link within the autopsy report and see images of the autopsy.*

It is crucial that any access and permission management system for sensitive images include the patient portal. While organizations may choose to apply default access to imaging studies, patients should have the ability to state and define their own preferences. For example, an organization may provide patients open access to images containing nudity (Clinical use cases # 1, 2, 4, 6, 7, 8, 9, 11, and 13) and limited access to gruesome images (Clinical use cases #8). In this scenario, a patient with body dysmorphic disorder (Clinical use cases, 3, 4, 7, 10, and 12) may choose to restrict their own access to images containing nudity as these images may cause emotional distress. It is important to state that this user preference could be changed later.

Patients should have the ability to set viewing preferences for those whom they entrust with proxy access to their medical records. For example, an elderly parent may provide their adult child with proxy access to their medical records so that the child can help their parent navigate the healthcare system. In this situation, the parent may choose to restrict access to images containing nudity (Clinical use case #11). Like providers, the proxy should also be able to enhance their level of access based on their own preferences. Note that this level of access should not be greater than that allowed by the patient.

Organizations should also be able to apply default permissions related to proxy access. This type of default permission should be rule-based. This would enable parents of young children to have open access to images of their child containing nudity. However, this permission could automatically change to restricted access when the child is an adolescent (Clinical use case #9). This type of default permission would protect proxy users from easily viewing gruesome images (Clinical use case #12).

Finally, we believe that patients should have the ability to add (or petition to add) a sensitive tag to an imaging study (Clinical use case 8). This ability would provide the patient autonomy and recognizes that the perceived sensitivity of content varies between individuals and can change with time.

## Image Sharing

### Clinical Use Case #13

*A 56-year-old man with melanoma is seeking a second opinion with another dermatologist. Initially, dermatoscope photographs of the melanoma and whole-body PET-CT images are sent to the second organization. While reviewing the patient’s record, the second dermatologist notices that whole-body mole mapping photographs were not sent because they were tagged as sensitive, containing nudity.*

Image sharing is a special use case that requires additional mention. Today, because few organizations systematically capture and store medical photography, there is little cross-institutional sharing of sensitive images. As enterprise imaging expands, this will change.

In the current model, when a patient requests that their imaging studies be sent to another hospital for a second opinion, many organizations send all imaging studies. This works because most image sharing is related to radiological images. However, in the future, state clinical photographs will be interspersed with radiologic images and other specialty-based images. This means that many imaging studies, including those unrelated to the second opinion request, are sent to the second organization (Clinical use case #6).

To enable modern image sharing, two actions are needed. First, images need to be able to be grouped so that relevant images can be shared. The HIMSS-SIIM Enterprise Imaging Community has previously identified body part as one of the key tags to enable advanced image sharing [8]. Other potential tags could include specialty or diagnosis. However, each of these tags is limited in their scope. For example, in Clinical use case #6, if only gastroenterology images were shared, other relevant imaging studies obtained within radiology and pathology would not be included. Additionally, because the diagnosis is not known at all time points of imaging, key imaging obtained prior to diagnosis would not be included. Manual selection of images to be shared is possible. However, this selection is time-consuming and often relies on the patient or clerical staff to make this determination.

The second action required to enable modern image sharing is the incorporation of patient preference. Patients should be able to specify the type of image sharing that they would like. This preference could be set and applied to all future image sharing or could apply only to a specific instance of image sharing. If this type of permission were enabled, we believe that four tiers of sharing are possible:

1. Open sharing: all images are shared, regardless of sensitivity tags.
2. Relevant sharing: sensitive images relevant to the body part, condition, or specialty are shared.
3. Specific image sharing: only specific imaging studies are shared, as specified by the patient.
4. Restricted sharing: only images that do not contain a sensitive tag are shared.

To supplement this model, providers at the second organization should be able to see that other imaging studies exist (Clinical use case #13). If these additional imaging studies were not transferred, the providers at the second organization should be able to request access to the images. Patients should be able to grant this access, automatically initiating image transfer, via the patient portal.

## Interoperability

The modern electronic medical record has enabled cross-institutional note sharing [9, 10]. As this functionality has expanded, reference-quality key images have been included for notes related to imaging results. When clinical photographs become incorporated into the imaging record, these images will be included as part of note sharing. There are currently no restrictions or protections related to sensitive images. Again, we believe that a tier-based approach is needed.

We encourage the electronic medical record vendors to add protections such as image-blurring and enhanced auditing for all imaging studies tagged as sensitive. Additionally, we believe that the default access to sensitive images obtained and stored at another institution should be limited. This access permission would enable providers to view the images and related note but would require additional entry of their password, highlighting the sensitive nature of the images. Patients may choose to further enhance this level of security as described above.

While we believe that patients should have the right to define their preferences for this type of image sharing, we recognize that restrictions may constitute information blocking as defined in the 21st Century Cures Act [11]. Continued advocacy is needed to incorporate patient preferences.

## Conclusion

Sensitive images represent a new challenge for imaging informaticists. These imaging studies are often defined by the presence of nudity, gruesome content, or forensic information. While this type of content can guide the standard application of study tags, sensitivity should also reflect the desires of the patient and the preferences of the people viewing the images (patient proxy users and healthcare providers). Currently, standards do not exist to provide adequate sensitive study tags. Because of this, software applications are not able to provide patients and end users with the protections that they need. The HIMSS-SIIM Enterprise Imaging Community has identified many challenges related to sensitive images. While some recommendations can be implemented today, the community believes that continual action is needed to develop and implement long-term solutions to protect the patients who entrust us with their care.
